# Can patient self-evaluation of functional status be used for evaluation of impairment of motor function in Guillain-Barré syndrome? Mapping clinician- and patient-reported outcomes in a phase 3 study of eculizumab in Japan

**DOI:** 10.3389/fneur.2025.1463938

**Published:** 2025-02-12

**Authors:** Antoine Regnault, Angély Loubert, Stéphane Quéré, Qun Lin, Glen Frick, Hirokazu Ishida, Yuko Abeta, Helene Chevrou-Severac

**Affiliations:** ^1^Modus Outcomes, A THREAD Company, Lyon, France; ^2^Alexion, AstraZeneca Rare Disease, Boston, MA, United States; ^3^Alexion, AstraZeneca Rare Disease, Tokyo, Japan; ^4^Alexion, AstraZeneca Rare Disease, Baar, Switzerland

**Keywords:** Guillain-Barré syndrome, clinician-reported outcomes, patient-reported outcomes, treatment outcomes, disease progression, eculizumab

## Abstract

**Background and purpose:**

Guillain-Barré syndrome (GBS) is an autoimmune neurological disorder characterized by muscle weakness. In clinical trials, treatment benefit and disease severity are typically measured using clinician-reported outcome measures like the Hughes Functional Grading Scale (HFGS). However, patient-reported outcome measures, such as the Rasch-built Overall Disability Scale (R-ODS) may provide additional insight into the patient experience during treatment. In this study, exploratory analyses of clinical trial data were performed to investigate how existing clinician-reported outcomes and patient-reported outcomes can help to assess disease progression by providing an accurate measurement of functional status.

**Methods:**

Data were collected as part of a phase 3 study to assess the safety and efficacy of eculizumab in patients in Japan with severe GBS. The association between HFGS score and R-ODS total centile score (linear measure of limitations; 0, most severe activity and social participation limitations and 100, no limitations) was assessed using the Spearman rank-order correlation coefficient. Threshold values of R-ODS total centile score that could differentiate between patients with an HFGS score of ≤ 1 and > 1 were determined using receiver-operating characteristic curve analyses and mapping (Rasch measurement theory). A triangulation approach was used to establish a proposed value for R-ODS total centile score equivalent to an HFGS score of ≤ 1 or > 1.

**Results:**

Overall, 57 patients were included in this analysis. These exploratory analyses revealed good correlation between R-ODS total centile and HFGS scores. Using the Rasch model, mapping of HFGS to R-ODS scores showed a good fit. Evaluation of the R-ODS threshold that could approximate the functional motor symptom categories based on HFGS (score of 0 or 1) revealed a range of values from 60 to 80. Based on a trial sample, a threshold of 60 was found to have 100% sensitivity and 87% specificity at week 4, and 93.8% sensitivity and 77.8% specificity at week 24.

**Conclusion:**

This study established thresholds for the R-ODS total centile score that could approximate classification of functional impairment in GBS based on the HFGS score. Given that the R-ODS reflects the patient perspective, it may be used to capture a more complete picture of GBS severity.

## 1 Introduction

Guillain-Barré syndrome (GBS) is an autoimmune neurological disorder with an annual global incidence of approximately 1–2 per 100,000 person-years (1, 2). GBS is the most common cause of flaccid tetraplegia worldwide, and its symptoms are characterized by muscle weakness, which typically starts in the distal lower extremities and progresses upwards over a period of hours or days until the arms and facial muscles also become affected (3, 4).

In mild cases, GBS symptoms may spontaneously decrease, but severe forms often require ventilator support, with recovery taking months or years (1). Even if treated with standard of care (plasma exchange or intravenous immunoglobulin), up to 20% of patients cannot walk independently at 1 year after disease onset, and approximately 3–10% of patients will die of disease-related complications (1, 5, 6). In addition to incomplete recovery of motor function, many patients develop severe weakness and have a long disease course, often with pain and fatigue, which results in significant social and physical impacts (7, 8).

Because impaired motor function is the main symptom of GBS, treatment benefit in clinical studies has typically been evaluated using clinical outcome assessments that measure functional status. These include clinician-reported outcomes (ClinROs) such as the Hughes Functional Grading Scale (HFGS) and the Overall Neuropathy Limitations Scale (ONLS). In parallel, the use of patient-reported outcomes (PROs) to support the definition of clinical study endpoints has gained a lot of interest in recent years (9). This includes the Rasch-built Overall Disability Scale (R-ODS), which reflects the patient perspective of disease status, measuring activity and social participation limitations in patients with neurological conditions such as GBS (9–12). The psychometric properties of the R-ODS have been previously assessed, including in participants with GBS (10), and has been used previously in clinical trial endpoints (12–15).

One notable benefit of the R-ODS, and of PROs in general, is that they allow for more frequent assessment because they do not require physical examination and they can be completed via electronic devices. As such, they represent a potentially useful tool for capturing disease status between regular clinic visits. For PRO measures to be useful in evaluating disease status in patients with GBS, it is essential to understand how they relate to the measures used for clinical evaluation. In this context, studying how the R-ODS relates to the HFGS is an important question to allow characterization of GBS disease status using PROs.

In a recent phase 3 trial evaluating the efficacy and safety of eculizumab in patients with severe GBS in Japan (NCT04752566) (16) the HFGS was used to measure treatment outcomes, with the primary endpoint of the trial defined as time to first reaching an HFGS score of ≤ 1. This was considered an adequate measure of disease recovery because an HFGS score of 0 is considered a healthy state and a higher score corresponds to more severe symptoms (i.e., 1, slight clinical signs and symptoms; 2, able to walk 10 meters or more without assistance, but not run; 3, able to walk 10 meters with help) (17). ONLS data were also collected to support a secondary endpoint, in addition to PRO data (R-ODS and EQ-5D-5L). Collection of ClinRO and PRO data in the trial provided an opportunity to generate new evidence on the assessment of the signs and symptoms of GBS progression from both the clinician and patient perspective.

In this study we performed exploratory analyses of the trial data to investigate how existing ClinRO and PRO measures can help in documenting the progression of GBS by providing an accurate measure of functional status. To address this, the specific objectives were to: (1) assess whether the R-ODS total centile score can provide a proxy for the identification of symptomatic resolution of functional impairment by the HFSG, whenever the latter cannot be assessed; and (2) explore the relationship between HFGS and R-ODS to consolidate our understanding of GBS progression from a clinician and patient perspective.

## 2 Materials and methods

### 2.1 Study design

These exploratory analyses were conducted using data collected in a phase 3, prospective, multicenter, placebo-controlled, double-blind, randomized study assessing the safety and efficacy of eculizumab in patients in Japan with severe GBS. Severe GBS was defined as an HFGS score progressively deteriorating to 3 or 4/5 within 2 weeks from the onset of weakness due to GBS (NCT04752566) (16). Eligible patients were randomized (2:1) to receive IV eculizumab (900 mg weekly for 4 weeks; a supplemental dose of 600 mg was given with the first dose on day 1) or placebo. All patients received concomitant intravenous immunoglobulin therapy as per standard of care (400 mg/kg body weight daily for 5 days).

The schedule of clinical outcome assessments is shown in Supplementary Table 1. HFGS score was collected at all trial visits, including screening, treatment period (weeks 0, 1, 2, 3, and 4), follow-up period (weeks 5, 6, 8, 12, 16, 20, and 24) and early termination visit. R-ODS score was collected at weeks 0 and 4 during the treatment period, weeks 8, 12, and 24 during the follow-up period and at the early termination visit. ONLS score was collected at weeks 0, 1, 2, 3, and 4 during the treatment period, weeks 8, 12, and 24 during the follow-up period, and at the early termination visit. EQ-5D-5L mobility score was collected at week 0 during the treatment period, weeks 5 and 24 during the follow-up period, and at the early termination visit.

Analyses were performed using the full analysis set, defined as all patients who had received at least one dose of the study drug or placebo and had a baseline HFGS score and at least one post-baseline HFGS score. All analyses described here were performed blinded to treatment.

### 2.2 Clinician-reported and patient-reported outcome assessments

#### 2.2.1 Hughes functional grading scale

The HFGS is a ClinRO scoring system used to assess the functional status of patients with GBS and was originally developed in 1978 by Hughes et al. (17). On the scale, a score of 0 indicates a healthy state, 1 indicates minor symptoms and capable of running, 2 indicates being able to walk 10 meters or more without assistance but being unable to run, 3 indicates being able to walk 10 meters across an open space with help, 4 indicates that the patient is bedridden or chairbound, 5 is applied when the patient requires assisted ventilation for at least part of the day, and a score of 6 indicates death.

#### 2.2.2 Rasch-built overall disability scale

The R-ODS is a 24-item self-administered PRO scale that specifically captures activity and social participation limitations in patients with neurological conditions such as GBS (10). Items included in the list are (in order of those difficult to perform with low limitations to those difficult to perform with high limitations): run, dance, remain standing for several hours, carry and put down heavy objects, walk up to 0.6 miles, use public transport, walk one flight of stairs, shopping, avoid obstacles while walking, make a sandwich, catch an object, visit primary physician, do washing up, bend and pick up object, move chair, wash lower body, toilet, shower, wash upper body, dress upper body, turn key in lock, reading, eating, and brushing teeth. Each item or activity on the scale is scored according to the patient’s self-evaluation of their usual ability to perform a task as follows: 0 = not possible to perform; 1 = possible, with some difficulty; and 2 = possible, without difficulty. The responses to the 24 items are summed in a total raw score ranging from 0 to 48. The R-ODS was developed within the framework of Rasch measurement theory (RMT), which means that the sum of the item responses can be transformed to a total centile score, which provides a linear measure of the level of activity and limitations. The total centile score ranges from 0 (most severe activity and social participation limitations) to 100 (no activity or social participation limitations) and can be visualized using a nomogram. A lower patient total score (raw or transformed to centiles) indicates more limitations on activities of daily living for patients.

#### 2.2.3 Overall Neuropathy Limitations Scale

The ONLS is a ClinRO checklist for observing current symptoms experienced by patients in their hands or arms (numbness, tingling, or weakness) and legs (e.g., difficulty running or climbing stairs, difficulty with walking) (18). The ONLS is completed by specialized clinicians who examine patients to determine their ability to perform specific arm- or leg-related activities, such as washing/brushing their hair or running/climbing stairs. One item relates to arm function (ranging from 0 to 5) and another item relates to leg function (ranging from 0 to 7). The ONLS total score is calculated by summing the arm and leg item scores, with total scores ranging from 0 to 12, with 0 meaning no movement disability in a patient’s legs and arms and 12 meaning maximum disability. A higher ONLS total score indicates higher neuropathy limitations in that patient’s limbs.

#### 2.2.4 EQ-5D-5L

The self-administered EQ-5D-5L is a two-part PRO instrument measuring quality of life (QoL), which allows the evaluation and comparison of health status across outcome areas (19). The first section of the EQ-5D questionnaire consists of five questions related to five dimensions of health-related QoL: mobility, self-care, usual activities, pain/discomfort, and anxiety/depression. This produces a five-digit health state profile that represents the level of reported problems (for the following five levels [score in brackets]: no [1], slight [2], moderate [3], severe [4], or unable to/extreme problems [5]) for each of the five dimensions. These health states are converted into a single weighted health utility score, with a value between 0 (worst health) and 1 (perfect health). The second section of the questionnaire, a visual analog scale (VAS), records the respondent’s own assessment of their health status on a scale from 0 to 100, where 100 is the best health you can imagine and 0 is the worst health you can imagine. In the current analysis, only the mobility domain in the first section of the questionnaire was used.

### 2.3 Statistical analyses

Because the research questions were independent of the question of treatment received, all analyses were performed on pooled treatment arms. Except for the RMT analyses, all data were analyzed using SAS software version 9.4 (SAS Institute Inc., Cary, NC, USA). RMT analyses were performed using RUMM software version 2030 (RUMM Laboratory Pty Ltd., Perth, Australia).

#### 2.3.1 Exploratory analysis of correspondence between HFGS-based categories and R-ODS-based categories

The association between HFGS score and R-ODS total centile score was assessed using the Spearman rank-order correlation coefficient. Distributions were compared using boxplots. Two approaches were then used to determine the value for R-ODS total centile score that could be used as a threshold to differentiate between patients with an HFGS score of ≤ 1, which signals that the patient’s GBS symptoms are resolving to normal or slight, and patients with an HFGS score > 1.

First, receiver-operating characteristic (ROC) curve analysis was performed in the full analysis set at baseline, week 4, and week 24. The area under the ROC curve was calculated to determine the ability of the R-ODS total centile score to discriminate between the two categories of patients defined by HFGS; this value was then used as an indicator of the association between these outcome assessments. The R-ODS total centile score that maximized the separation between the groups was estimated by the smallest sum of squares of 1 − sensitivity and 1 − specificity (20), and was considered as an estimate for an R-ODS threshold.

Second, mapping was performed using a two-step RMT approach that involved analyzing the R-ODS items with the polytomous Rasch model and then mapping HFGS to the R-ODS items. The Rasch model is a probabilistic model, which considers that the responses to the items depend on the patients’ assessment of symptom severity and task difficulty. The RMT analysis was performed using all available data (pooled weeks 0/baseline, 4, 8, 12, and 24) to maximize the sample size (‘stacked’ RMT analysis) (21). A threshold for categorization based on the R-ODS total centile score was determined using the following approaches within the RMT analysis: (1) using the estimated threshold parameter between HFGS rating (1 and 2); (2) using the item characteristic curve of the HFGS score; and (3) equating the R-ODS total centile score and HFGS score.

The results of the above two approaches were considered together in a triangulation exercise to propose a threshold for the R-ODS total centile score that could be equivalent to an HFGS score of| ≤ 1. To compare the categorization of patients using the HFGS or the R-ODS, the proportion of patients that reached an R-ODS total centile score below the defined threshold was calculated at weeks 4 and 24 and compared with the proportion of patients who reached an HFGS score of ≤ 1 at the same visit. The sensitivity and specificity of the R-ODS-based categories were also calculated.

#### 2.3.2 Characterization of symptomatic severity of GBS by HFGS, ONLS, and EQ-5D-5L mobility domain

To estimate the parameters for all items of the R-ODS, mapping was performed using the two-step RMT approach that involved analyzing the R-ODS items with the Rasch model and then mapping HFGS scores, ONLS items, and EQ-5D-5L mobility scores to the R-ODS items. With this analysis, the R-ODS item parameter estimates were fixed and those from the HFGS, ONLS, and EQ-5D-5L mobility domain were estimated. The relative ranges of the R-ODS metrics covered by these ClinRO measures were evaluated, and the order of items along the continuum was assessed to provide a characterization of the symptomatic severity of GBS.

#### 2.3.3 Assessment of the relationship between HFGS and EQ-5D-5L mobility score

The strength of the relationship between HFGS and EQ-5D-5L mobility score at baseline was assessed using cross-tabulation and Spearman rank-order correlation.

### 2.4 Ethics and approvals

This study was reviewed and approved by an independent ethics committee (non-profit organization MINS Institutional Review Board, Tokyo, Japan, Approval ID: 200233).

## 3 Results

### 3.1 Patient characteristics

Detailed baseline patient demographic and clinical characteristics have been published previously (16). In brief, 57 patients were included in this analysis (*n* = 37 in the eculizumab treatment arm and *n* = 20 in the placebo group; Table 1). The characteristics were similar between treatment arms and the mean (SD) age was 56.6 (19.6) and 56.2 (18.3) years in the eculizumab and placebo groups, respectively.

**Table 1:** Baseline demographic and clinical characteristics.

Characteristic	Eculizumab + IVIg (*n* = 37)	Placebo + IVIg (*n* = 20)
Sex, *n* (%)		
Male	18 (48.6)	15 (75.0)
Female	19 (51.4)	5 (25.0)
Race, *n* (%)		
Asian	37 (100)	20 (100)
Age, years		
Mean (SD)	56.6 (19.6)	56.2 (18.3)
Median (IQR)	58.0 (35.0)	55.0 (24.0)
Minimum, maximum	19, 84	21, 84
Age category, years, *n* (%)		
18 to < 60	20 (54.1)	11 (55.0)
≥ 60	17 (45.9)	9 (45.0)
HFGS score, *n* (%)		
3^a^	6 (16.2)	3 (15.0)
4/5^b^	31 (83.8)	17 (85.0)
GBS subtype, *n* (%)		
AIDP	22 (59.5)	10 (50.0)
AMAN	8 (21.6)	3 (15.0)
Indeterminate	7 (18.9)	7 (35.0)

a HFGS score of 3 indicates progressive/deteriorating disease.

b HFGS score of 4 or 5 indicates stable or progressively deteriorating disease.

AIDP, acute inflammatory demyelinating polyneuropathy; AMAN, acute motor axonal neuropathy; GBS, Guillain-Barré syndrome; HFGS, Hughes Functional Grading Scale; IQR, interquartile range; IVIg, intravenous immunoglobulin; SD, standard deviation.

### 3.2 Exploratory analysis of correspondence between HFGS-based categories and R-ODS-based categories

**Figure 1:**
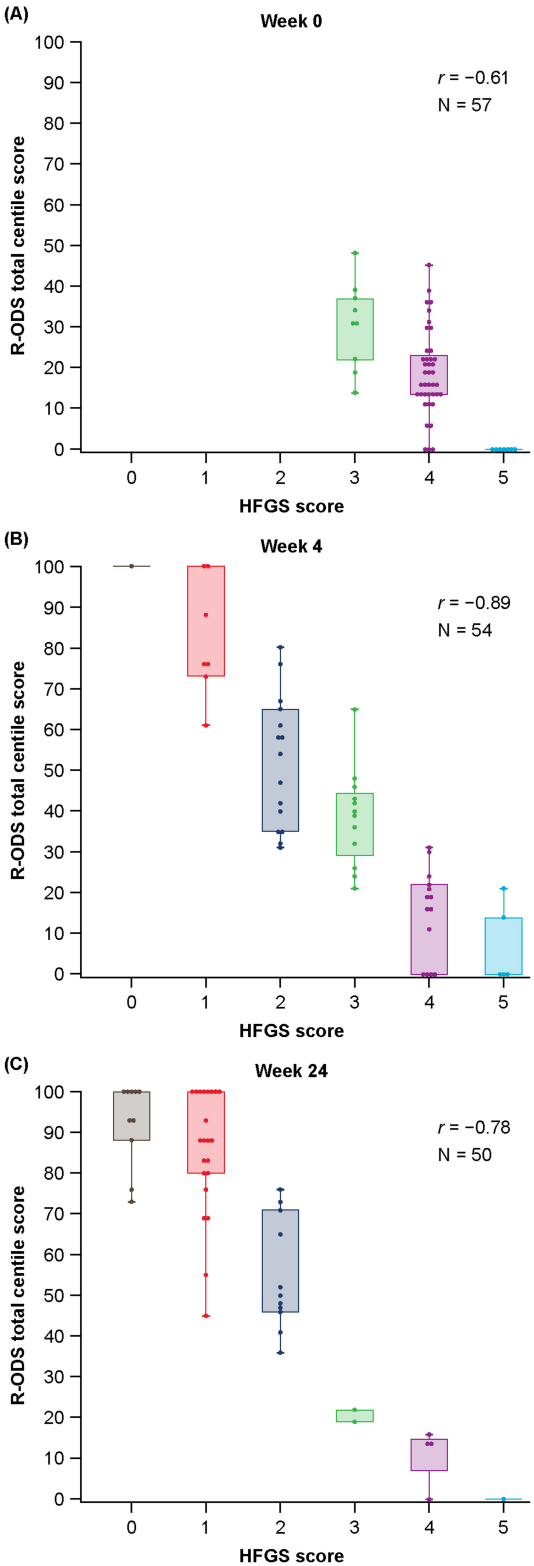
Distribution of R-ODS total centile scores with HFGS at **(A)** baseline, **(B)** week 4, and **(C)** week 24. HFGS, Hughes Functional Grading Scale; *r*, correlation coefficient; R-ODS, Rasch-built Overall Disability Scale.

**Figure 2:**
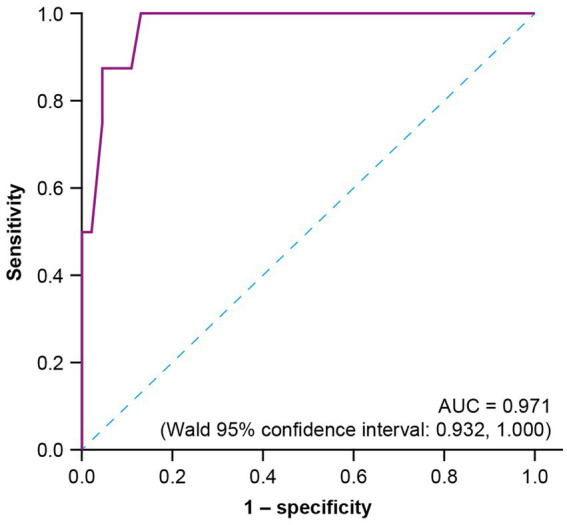
ROC curve analysis at week 4 to estimate the R-ODS threshold that could approximate the functional motor symptom categories based on HFGS functional grade. AUC, area under the curve; HFGS, Hughes Functional Grading Scale; ROC, receiver-operating characteristic; R-ODS, Rasch-built Overall Disability Scale.

Compared with baseline, a higher correlation between R-ODS total centile score and HFGS score was observed at all post-baseline timepoints (Figure 1). The post-baseline scores at week 4 (*r* = −0.89, *n* = 54) and week 24 (*r* = −0.78, *n* = 50) showed high correlation. A moderate correlation was observed at baseline (*r* = −0.61, *n* = 57) when all patients had an HFGS score of ≥ 3 (as per the inclusion criteria). ROC curve analysis to estimate the R-ODS threshold that could approximate the HFGS-based functional motor symptom categories showed a very high AUC of 0.971 (Wald 95% confidence interval: 0.932, 1.000) at week 4, demonstrating that the R-ODS score could discriminate between patients who had an HFGS score of ≤ 1 and those with a score of > 1 (Figure 2). The R-ODS threshold that maximized the separation (with equal weight for false positives and false negatives) between the two patient categories at week 4 was 60. Results were similar at other explored timepoints.

**Figure 3:**
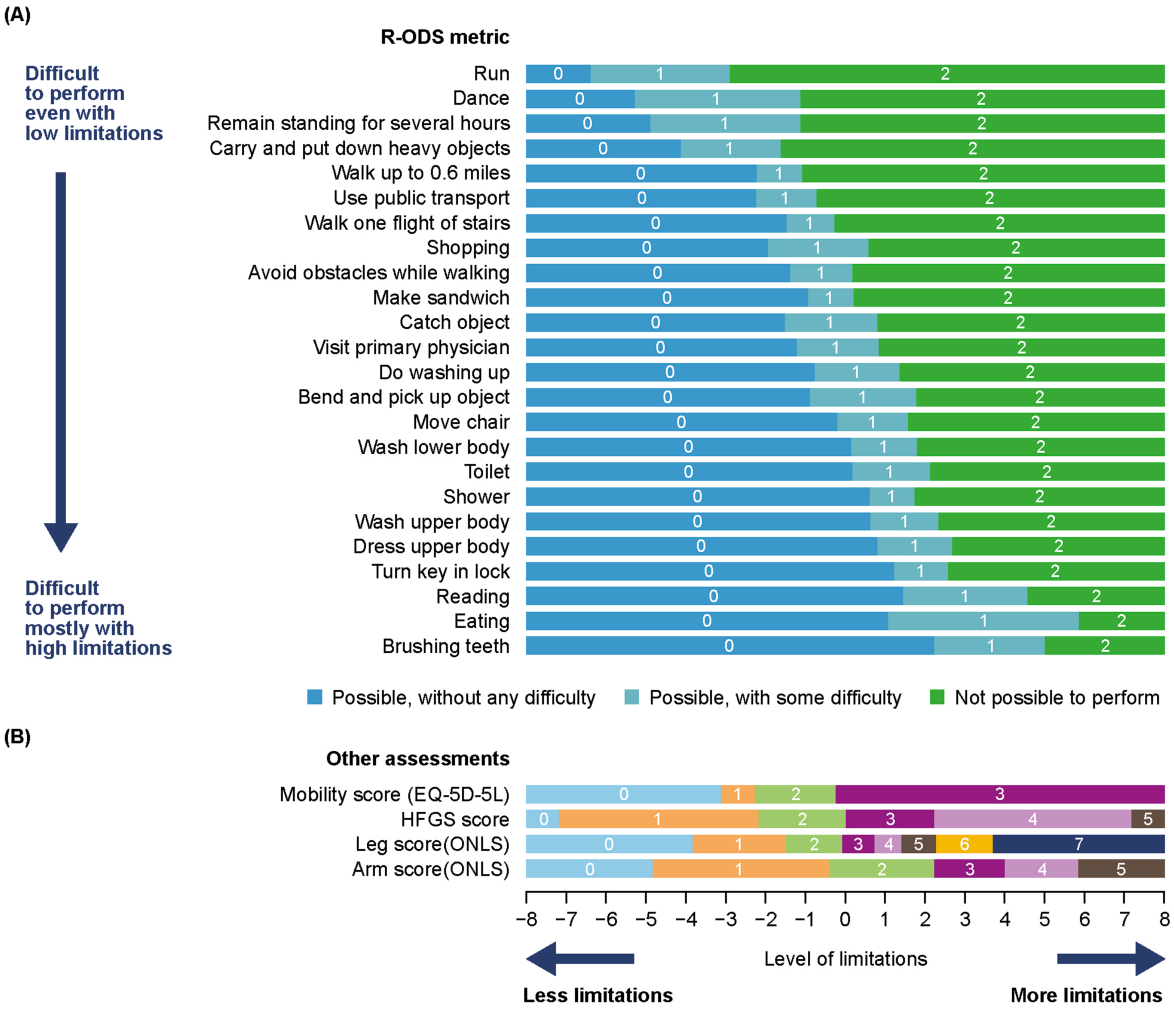
**(A)** RMT analysis of the R-ODS metric and **(B)** mapping of HFGS, ONLS, and EQ-5D-5L mobility results to the R-ODS^a^. The *x*-axis represents the measurement continuum of the level of limitation (metric obtained from the Rasch model, on a logit scale), with increasing levels from left to right. The *y*-axis shows each of the items in order of item severity from top to bottom. The color blocks indicate the response options most likely to be endorsed by participants depending on their symptom severity/level of limitation (based on the prediction probability to endorse each response option as per the Rasch model, depending on person location, on the same continuum). ^a^Compared with the raw score, the response options have been reversed here; with a score of 0 = possible without any difficulty, 1 = possible with some difficulty, and 2 = not possible. HFGS, Hughes Functional Grading Scale; ONLS, Overall Neuropathy Limitations Scale; RMT, Rasch measurement theory; R-ODS, Rasch-built Overall Disability Scale.

RMT analysis was performed using all available data (pooled weeks 0/baseline, 4, 8, 12, and 24); this showed a good fit of the R-ODS items to the Rasch model. There was a clear hierarchy of the response categories for the R-ODS items depending on the level of limitation experienced by patients (Figure 3A). The observed hierarchy of limitations in performing different tasks elicited by the Rasch model described a meaningful progression from running to brushing teeth.

**Figure 4:**
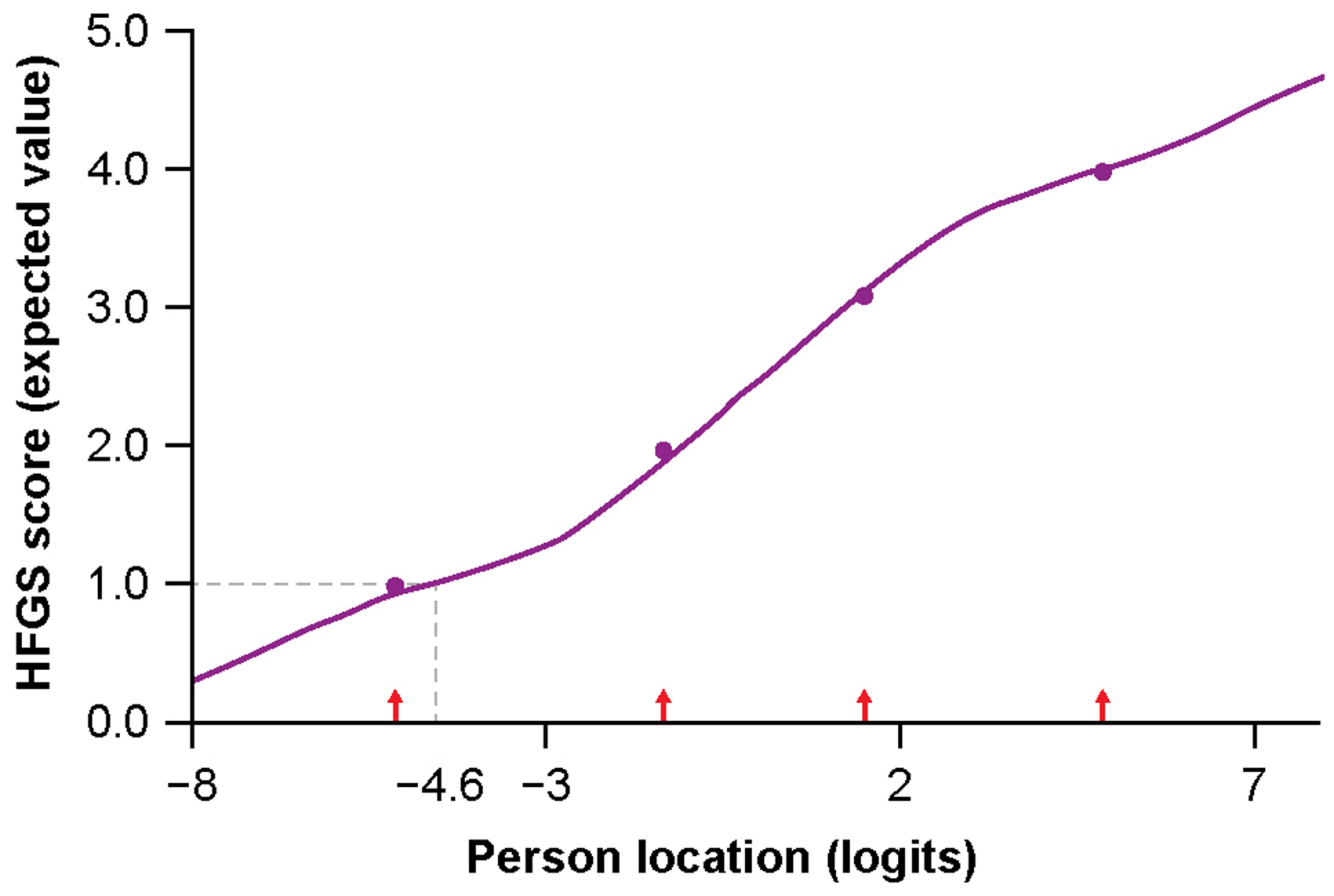
Item characteristic curve from mapping of HFGS score to the R-ODS items (leading to estimated threshold of 80 after linear transformation of person location). Dashed line indicates estimation of the R-ODS threshold, based on HFGS = 1 (leading to a threshold of 80 after linear transformation of the person location [in logits]). HFGS, Hughes Functional Grading Scale; R-ODS, Rasch-built Overall Disability Scale.

Using the Rasch model, the mapping of the HFGS to the R-ODS showed a good fit. Results showed low fit residuals (0.4) and a non-significant difference between the observed responses and those predicted from the Rasch model (*p* = 0.22), as visible on the item characteristic curve (Figure 4). Mapping of HFGS within the R-ODS showed that patients with an HFGS score of ≤ 1 would typically show, at worst, only difficulty with run, dance, remain standing for several hours, and carry and put down heavy objects (Figure 3B). Patients with an HFGS score of > 1 would typically show difficulty in more activities captured by the R-ODS (Figure 3B). Possible R-ODS thresholds equivalent to an HFGS score of ≤ 1 and > 1 ranged between 63 and 80, depending on the approach used (Table 2).

**Table 2:** Estimation of possible threshold for R-ODS total centile scores equivalent to HFGS categorization, based on RMT mapping.

Method	HFGS	R-ODS total raw score	R-ODS metric (logit)	Equivalent transformed R-ODS total centile score	R-ODS total centile score
Threshold between HFGS 1 and 2	1 vs 2		−2.12	63	
Item characteristic curve of the HFGS	1		−4.60	80	
Equating between R-ODS score and HFGS	1	42	−3.21	71	73
	2	31	−0.94	55	54
	3	17	1.10	41	36
	4	5	3.36	25	19

HFGS, Hughes Functional Grading Scale; RMT, Rasch measurement theory; R-ODS, Rasch-built Overall Disability Scale.

The RMT framework allows exploration of the probability of observing different HFGS scores for a given patient depending on the R-ODS threshold used (Supplementary Figure 1). Considering an R-ODS threshold of 80, a patient would have a 92% chance of having an HFGS score of ≤ 1 and an 8% chance of having a score of > 1. When a threshold of 70 is considered, a patient with a score of 70 would have a 72% chance of having score of ≤ 1 and a 28% chance of having a score of > 1. For a threshold of 60, a patient with a score of 60 would have a 34% chance of having a score of ≤ 1 and a 66% chance of having a score of > 1.

A comparison of the categorization based on the HFGS and the three possible R-ODS thresholds in the trial sample found that an R-ODS threshold of 60 had a sensitivity of 100% and a specificity of 87.0% at week 4, and a sensitivity of 93.8% and a specificity of 77.8% at week 24. A threshold of 70 had a sensitivity of 87.5% and a specificity of 95.6% at week 4, and a sensitivity of 87.5% and a specificity of 83.3% at week 24. Finally, a threshold of 80 had a sensitivity of 50.0% and a specificity of 97.8% at week 4, and a sensitivity of 78.1% and a specificity of 100% at week 24 (Table 3).

**Table 3:** Comparison of HFGS categories and R-ODS thresholds.

R-ODS centile score	HFGS		R-ODS threshold sensitivity (%)	R-ODS threshold sensitivity (%)
	≤ 1, *n* (%)	> 1, *n* (%)		
Week 4				
≥ 60	8 (15)	6 (11)	100	87.0
< 60	0 (0)	40 (74)		
≥ 70	7 (13)	2 (4)	87.5	95.6
< 70	1 (2)	44 (81)		
≥ 80	4 (7)	1 (2)	50.0	97.8
< 80	4 (7)	45 (83)		
Week 24				
≥ 60	30 (60)	4 (8)	93.8	77.8
< 60	2 (4)	14 (28)		
≥ 70	28 (56)	3 (6)	87.5	83.3
< 70	4 (8)	15 (30)		
≥ 80	25 (50)	0 (0)	78.1	100
< 80	7 (14)	18 (36)		

HFGS, Hughes Functional Grading Scale; R-ODS, Rasch-built Overall Disability Scale.

### 3.3 Characterization of symptomatic severity of GBS by HFGS, ONLS, and EQ-5D-5L mobility domain

HFGS scores, ONLS items, and EQ-5D-5L mobility scores fitted the Rasch model “anchored” on the R-ODS, and showed clear hierarchy of the items over the continuum (Figure 3B). The impact of GBS as related to lower limb function, such as walking, climbing stairs, or running, was observed overall even for low levels of limitation. However, the impact of GBS as related to upper limb function, such as eating or brushing teeth, was observed only for higher levels of limitation.

### 3.4 Assessment of the relationship between HFGS and EQ-5D-5L mobility domain

A low association between HFGS and EQ-5D-5L mobility score was observed at baseline (Spearman rank-order correlation coefficient of 0.38; Table 4), when all patients had an HFGS score of ≥ 3 (as per the inclusion criteria), while the correlation at week 5 (0.83, *n* = 53) and week 24 (0.79, *n* = 50) was strong. All seven patients (100%) with an HFGS score of 5, and 39 of 41 patients (95.1%) with an HFGS score of 4, reported being unable to walk at baseline. Among patients with an HFGS score of 3 (*n* = 9), six (66.7%) reported in the EQ-5D-5L questionnaire being unable to walk at baseline, one (11.1%) reported severe problems walking about, and two (22.2%) reported moderate problems walking.

**Table 4:** Comparison of HFGS score and EQ-5D-5L mobility score.

Time point for EQ-5D-5L mobility score	*N*	Spearman rank-order correlation coefficient for HFGS score	
Baseline	57	0.38	
Week 5	53	0.83	
Week 24	50	0.79	
HFGS score at baseline			
Variable	3	4	5
Baseline, *n* (%)			
I am unable to walk about	6 (66.7)	39 (95.1)	7 (100.0)
I have severe problems in walking about	1 (11.1)	1 (2.4)	0 (0.0)
I have moderate problems in walking about	2 (22.2)	0 (0.0)	0 (0.0)
Missing	0 (0.0)	1 (2.4)	0 (0.0)

HFGS, Hughes Functional Grading Scale.

## 4 Discussion

This study established a strong association between the ClinRO and PRO assessments of functional limitation in patients with GBS. The results from this study also demonstrated that the classification of symptomatic resolution of functional impairment in GBS based on the HFGS can be accurately mirrored using the R-ODS in our data. Additionally, our findings showed that the clinical measures commonly used to evaluate neurological disease severity, namely the HFGS and ONLS, could be mapped onto the functional impairment metric as defined by the R-ODS. Overall, the results suggest that, despite being analytically different measures (single rating vs multi-item score), assessing different perspectives (clinician vs patient), these measures share a common source of variation and can be considered as measuring strongly related concepts. Furthermore, the item distribution over the common continuum underlying the scales clearly delineates a meaningful metric in terms of both functional limitations (from running to brushing one’s teeth) and clinical presentation, reflecting the typical presentation and progression of GBS symptoms (3, 4). This strengthens the conclusion that mapping between these measurement scales may be a relevant approach.

This approach of using a PRO measure as a proxy for a ClinRO measure may offer a solution for assessing progression of GBS in between regular clinic visits, or in situations in which these are not possible, for example, during long-term extension trials or observational studies. Interestingly, previous research has compared the R-ODS to ClinROs, such as the ONLS, showing that it may be better at detecting meaningful changes over time in patients with GBS (9). Our analyses were cross-sectional and did not compare the longitudinal performance of the ClinRO and PRO measures, so this conclusion cannot be confirmed in this case. However, the cross-sectional mapping of the instruments in our analyses illustrated that the R-ODS items provide good coverage of the metrics commonly assessed in GBS (i.e., they can be used to measure patient limitations through different levels of disease severity), while both ClinRO measures provide less granular coverage, which may explain the ability of the R-ODS to detect change better, as observed in previous studies (9). This greater granularity is made possible by the higher possible number of values for the multi-item R-ODS score compared to the single rating HFGS.

Once the correlation between the HFGS and the R-ODS had been demonstrated, a further objective of these analyses was to establish whether there is an R-ODS threshold that could effectively approximate the resolution of functional motor symptoms based on the HFGS score ≤ 1. Unsurprisingly, the various statistical analyses did not lead to a single estimate for this value; the R-ODS scores of interest typically ranged between 60 and 80, with the most reasonable range appearing to be between 63 and 73 for which an HFGS score of ≤ 1 is equivalent to an R-ODS score ≥ the threshold. This wide range of estimates reflects the uncertainty associated with statistical analyses, and the decision on the best R-ODS threshold to define symptomatic resolution will likely depend on the context and the risk that one is willing to take regarding incorrect allocation of symptom resolution in patients. As a result, in clinical and observational studies, sensitivity analyses using a range of thresholds would be recommended.

It should be noted that our analyses were conducted using data from a single study that included 57 patients and were exploratory in nature. Any estimation of statistics based on such a small sample is associated with uncertainty, and the estimates resulting from these analyses (thresholds for categorization, sensitivity, and specificity, etc.) should be considered with caution. Even though data were collected for each patient at multiple timepoints for inclusion in the analysis, it will be important to validate these results in a larger sample. Additionally, the sample was from a clinical trial in Japanese patients and so may not be representative of the wider GBS population. Further research in a broader patient population is warranted in addition to extending this study to other neurological diseases. It is worth noting that these analyses were cross-sectional and so although they characterize the severity of GBS, which could align with disease progression, longitudinal patient trajectories were not explored.

Further research is required to validate the results of this study; however, this study has demonstrated that it may be possible to use the R-ODS total centile score as an additional tool to evaluate the severity of GBS and resolution of functional limitations in situations where HFGS score collection is not possible. This approach may also allow for more frequent assessment between regular clinical visits, thus providing a more complete picture of changes over the course of the disease. Results from this research provide additional context for interpreting the R-ODS score, including correspondence with the HFGS score, which might be useful when the latter is not available for a patient at a given assessment. Of note, the goal of this research is not to suggest that the R-ODS total centile can act as a replacement for the HGFS score; however, this approach may be particularly useful for studies such as long-term follow-up studies for which more frequent collection of PRO data is beneficial. Using the R-ODS score in GBS studies is also conditional on demonstrating good measurement performance. Of note, supportive psychometric evidence for the R-ODS score has been generated in previous research, but areas such as longitudinal measurement properties (group-level responsiveness) remain to be fully documented (9, 22). Overall, the functional status of patients with GBS can be better characterized with items in the HFGS and the ONLS that capture upper and lower limb function using a common metric of activity and social participation limitations defined by the R-ODS, than would be possible by using the HFGS or ONLS alone. Improved methods for characterizing the signs and symptoms of GBS through different stages of the disease would be of benefit for clinical research, because they can contribute to more accurate detection of meaningful benefits of new treatments in clinical trials while also providing tools to support better clinical management of GBS, which may ultimately lead to improved outcomes for patients.

## Data Availability

Alexion, AstraZeneca Rare Disease will consider requests for disclosure of clinical study participant-level data provided that participant privacy is assured through methods like data de-identification, pseudonymization, or anonymization (as required by applicable law), and if such disclosure was included in the relevant study informed consent form or similar documentation. Qualified academic investigators may request participant-level clinical data and supporting documents (statistical analysis plan and protocol) pertaining to Alexion-sponsored studies. Further details regarding data availability and instructions for requesting information are available in the Alexion Clinical Trials Disclosure and Transparency Policy at https://www.alexionclinicaltrialtransparency.com/data-requests/.
