# Supplementary material for: Can patient self-evaluation of functional status be used for evaluation of impairment of motor function in Guillain-Barré syndrome? Mapping clinician- and patient-reported outcomes in a phase 3 study of eculizumab in Japan
Source: Front Neurol. 2025 Feb 12;16:1463938. doi: 10.3389/fneur.2025.1463938 (PMC11908375; doi:10.3389/fneur.2025.1463938)
Supplement: Supplementary file 1 [file Supplementary_file_1.docx]

**Can patient self-evaluation of functional status be used for evaluation of impairment of motor function in Guillain-Barré syndrome? Mapping clinician- and patient-reported outcomes in a phase 3 study of eculizumab in Japan**

Antoine Regnault^1^, Angély Loubert^1^, Stéphane Quéré^1^, Qun Lin^2^, Glen Frick^2^, Hirokazu Ishida^2^, Yuko Abeta^2^, Helene Chevrou-Severac^2^

^1^Modus Outcomes, A THREAD Company, Lyon, France

^2^Alexion, AstraZeneca Rare Disease, Boston, MA, USA

**Supplementary material**

**SUPPLEMENTARY FIGURE 1** Probability of HFGS score based on R-ODS threshold of (A) 80, (B) 70, or (C) 60


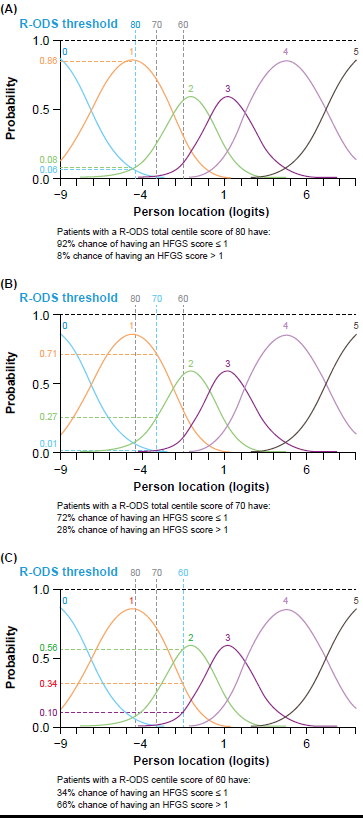


HFGS, Hughes Functional Grading Scale; R-ODS, Rasch-built Overall Disability Scale.

Legend: Numbers above curves indicate the HFGS for which the curve presents the probability of the patient being categorized with that score, depending on their level of GBS symptoms severity (person location, in logits).

**SUPPLEMENTARY TABLE 1** Schedule of clinical outcome assessments

|  | Screening | Treatment period | | | | | Follow-up period | | | | | | | ET |
| --- | --- | --- | --- | --- | --- | --- | --- | --- | --- | --- | --- | --- | --- | --- |
|  | V1 | V2 | V3 | V4 | V5 | V6 | V7 | V8 | V9 | V10 | V11 | V12 | V13 |  |
|  |  | W0 | W1 | W2 | W3 | W4 | W5 | W6 | W8 | W12 | W16 | W20 | W24 |  |
| HFGS | X | X | X | X | X | X | X | X | X | X | X | X | X | X |
| R-ODS |  | X |  |  |  | X |  |  | X | X |  |  | X | X |
| ONLS |  | X | X | X | X | X |  |  | X | X |  |  | X | X |
| EQ-5D-5L |  | X |  |  |  |  | X |  |  |  |  |  | X | X |

ET, early termination; HFGS, Hughes Functional Grading Scale; ONLS, Overall Neuropathy Limitations Scale; R-ODS, Rasch-built Overall Disability Scale; V, visit; W, week.
